# Navigating and expanding the roadmap of natural product genome mining tools

**DOI:** 10.3762/bjoc.18.178

**Published:** 2022-12-06

**Authors:** Friederike Biermann, Sebastian L Wenski, Eric J N Helfrich

**Affiliations:** 1 Institute for Molecular Bio Science, Goethe University Frankfurt, Max-von-Laue Str. 9, 60438 Frankfurt am Main, Germany; 2 LOEWE Center for Translational Biodiversity Genomics (TBG), Senckenberganlage 25, 60325 Frankfurt am Main, Germany

**Keywords:** genome mining, natural product biosynthesis, non-canonical pathways, PKS, NRPS, RiPP

## Abstract

Natural products are structurally highly diverse and exhibit a wide array of biological activities. As a result, they serve as an important source of new drug leads. Traditionally, natural products have been discovered by bioactivity-guided fractionation. The advent of genome sequencing technology has resulted in the introduction of an alternative approach towards novel natural product scaffolds: Genome mining. Genome mining is an in-silico natural product discovery strategy in which sequenced genomes are analyzed for the potential of the associated organism to produce natural products. Seemingly universal biosynthetic principles have been deciphered for most natural product classes that are used to detect natural product biosynthetic gene clusters using pathway-encoded conserved key enzymes, domains, or motifs as bait. Several generations of highly sophisticated tools have been developed for the biosynthetic rule-based identification of natural product gene clusters. Apart from these hard-coded algorithms, multiple tools that use machine learning-based approaches have been designed to complement the existing genome mining tool set and focus on natural product gene clusters that lack genes with conserved signature sequences. In this perspective, we take a closer look at state-of-the-art genome mining tools that are based on either hard-coded rules or machine learning algorithms, with an emphasis on the confidence of their predictions and potential to identify non-canonical natural product biosynthetic gene clusters. We highlight the genome mining pipelines' current strengths and limitations by contrasting their advantages and disadvantages. Moreover, we introduce two indirect biosynthetic gene cluster identification strategies that complement current workflows. The combination of all genome mining approaches will pave the way towards a more comprehensive understanding of the full biosynthetic repertoire encoded in microbial genome sequences.

## Introduction

**Figure 1:**
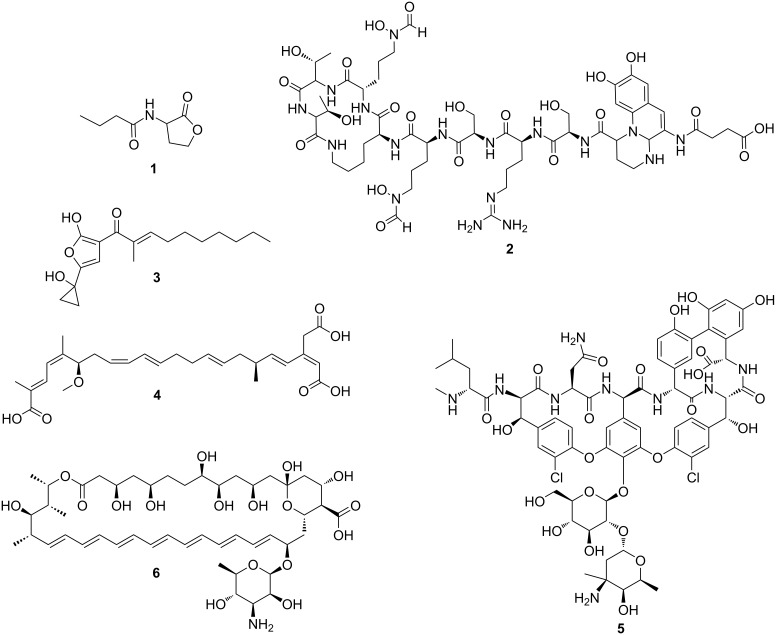
Examples of prominent natural products: *N*-butyrylhomoserine lactone (**1**), pyoverdin (**2**), malleicyprol (**3**), bongkrekic acid (**4**), vancomycin (**5**), and amphotericin B (**6**).

In 2002, the genome sequences of the model actinomycete *Streptomyces coelicolor* A3(2) [1] and the producer of the antiparasitic drug avermectin, *Streptomyces avermitilis* [2], were published. These index cases marked the transition from the pre- to the post-genomic era in microbial natural product (NP) research [3]. The introduction of next-generation sequencing technologies [4] has led to a constant decrease in sequencing costs [5]. As a result, the number of publicly available genome sequences has rapidly increased and paved the way for a completely new avenue: genome mining. Genome mining describes the targeted bioinformatic analysis of (meta-)genomes to identify gene clusters involved in the biosynthesis of NPs [3]. NPs have been shown to act as signaling metabolites (e.g., acylhomoserine lactones (**1**) [6]), siderophores (e.g., pyoverdines (**2**) [7]), virulence factors (e.g., malleicyprol (**3**) [8–10]), toxins (e.g., bongkrekic acid (**4**) [11]), antibacterial (e.g., vancomycin (**5**) [12]) or antifungal compounds (e.g., amphotericin B (**6**) [13]) (Figure 1). The identification of almost all clinically relevant antibiotics using bioactivity-guided fractionation approaches long before the beginning of the post-genomic era initiated the field of microbial NP research. In the "golden age" of antibiotic discovery from the 1940s to 1970s, microbes and especially bacteria have been identified as an almost untapped treasure trove for the discovery of bioactive NPs. For the longest time, researchers focused on a few talented NP producers, that have mainly been isolated from soil samples [14]. Since the low hanging fruits have been picked using traditional bioactivity-based workflows, this approach frequently results in the rediscovery of known metabolites. The introduction of genome mining revolutionized NP research and helped overcome the rediscovery problem frequently encountered using traditional approaches. Contrary to earlier estimations that were based on bioactivity-guided discovery strategies, mining microbial genomes revealed a much higher biosynthetic potential than initially anticipated [14]. *Streptomyces hygroscopicus* sp. XM201, for instance, harbors more than 50 putative biosynthetic gene clusters (BGCs), many of which are cryptic, i.e., BGCs for which the corresponding NPs have yet to be identified [15]. A problem when it comes to the characterization of the full biosynthetic potential of an organism is the fact that many BGCs are silent. Silent BGC are not expressed under standard laboratory cultivation conditions as they might lack a specific ecological clue for their expression. As a result, two types of approaches have been developed to unleash this hidden biosynthetic potential. Several pleiotropic (non-targeted, e.g., modifying culturing conditions) and pathway-specific (e.g., heterologous expression or in situ pathway activation) approaches have been developed to awaken silent biosynthetic pathways [16]. Most importantly, however, genome mining can prevent the time-consuming re-discovery of already known metabolites [14]. In-silico dereplication can be performed on two levels: First, BGCs identified by genome mining can be compared to characterized BGCs [17]. Second, in many cases NP core structures can be predicted from genome sequence information and the predicted structures can then be used to search in NP databases for identical or related compounds [18–19]. While the BGC-centric approach might be more accurate, it is limited by the number of characterized BGCs in publicly available databases. Since significantly more NPs than NP BGCs are characterized, the search space of known metabolites is significantly larger than that of experimentally verified BGCs [20]. The accuracy of the predicted core structures on the other hand might restrict the approach.

In this perspective, we will take a closer look at the most commonly used state-of-the-art genome mining tools, ranging from algorithms based on hard-coded rules to machine learning (ML)-based approaches with regard to the natural product biosynthetic principles they are most suited for. We focus on how the different genome mining tools identify BGCs and highlight their advantages and limitations. Moreover, we will showcase two potential strategies for the targeted identification of non-canonical pathways to chart the full biosynthetic potential encoded in bacterial genomes.

## Perspective

### Natural product biosynthetic principles

**Figure 2:**
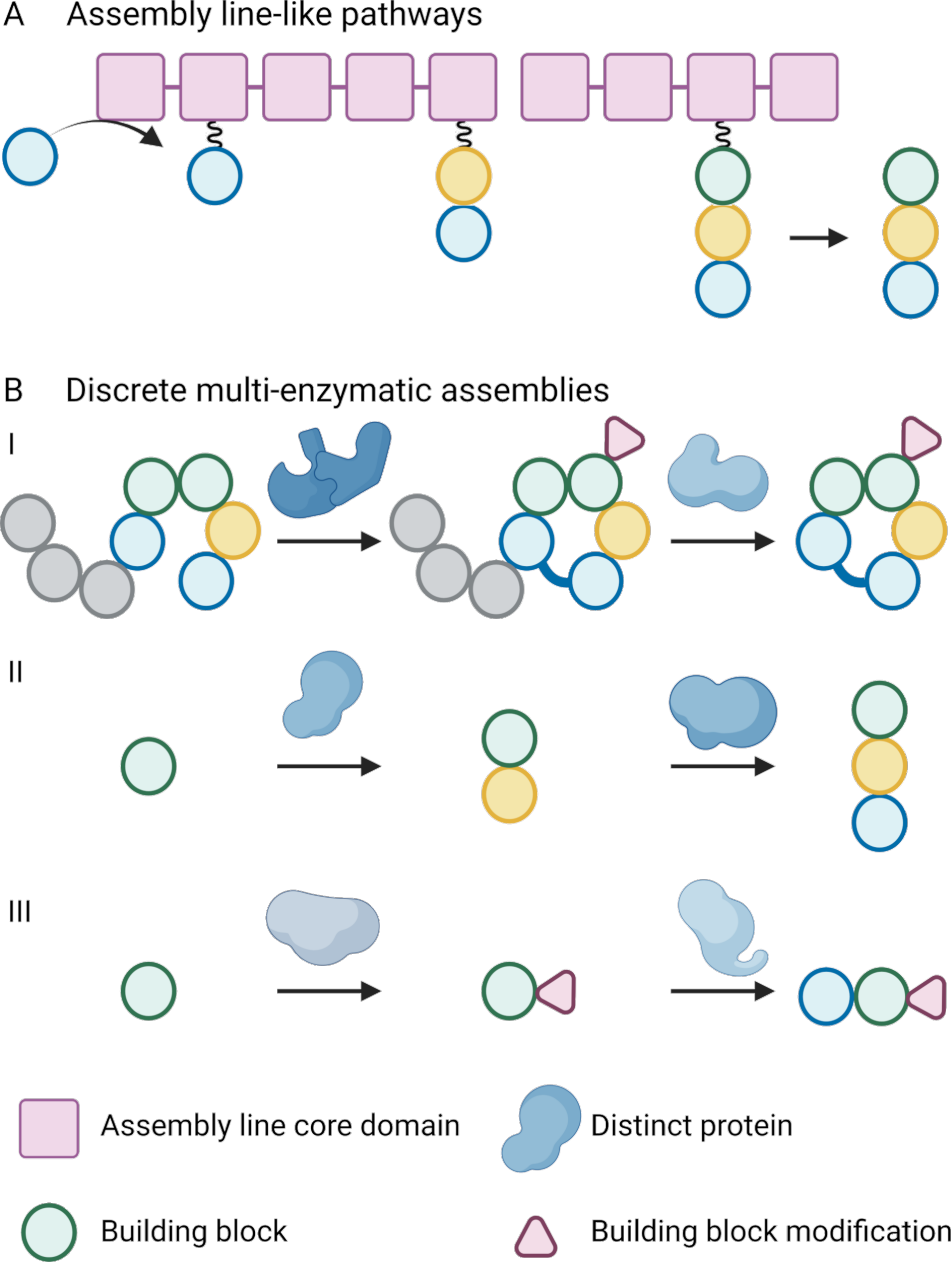
Biosynthetic principles of (A) assembly line-like pathways and (B) discrete multi-enzymatic assemblies. Assembly line-like pathways use large mega enzymes and generate NPs via the successive addition and/or modification of building blocks (e.g., non-ribosomal peptide biosynthesis) using conserved core domains. In discrete multi-enzymatic assemblies, distinct and mostly monofunctional enzymes catalyze the built-up of the NP scaffold and its decoration (e.g., in (I) ribosomally synthesized and post-translationally modified peptide, (II) terpene, or (III) alkaloid biosynthesis).

NPs are structurally highly diverse and can be divided into several classes depending on their biosynthetic concepts. NP biosynthesis follows two fundamentally different principles: NPs can either be produced in an assembly line-like fashion (Figure 2A) or by discrete, multi-enzymatic assemblies (Figure 2B). Discrete, multi-enzymatic assemblies utilize monofunctional enzymes for the consecutive build-up and decoration of a NP scaffold. In comparison to biosynthetic assembly lines, intermediates are not permanently covalently bound to carrier proteins in discrete, multi-enzymatic assemblies. In both biosynthetic principles, the NP backbone is first assembled by core enzymes and then further modified by tailoring enzymes that decorate the NP scaffold.

**Figure 3:**
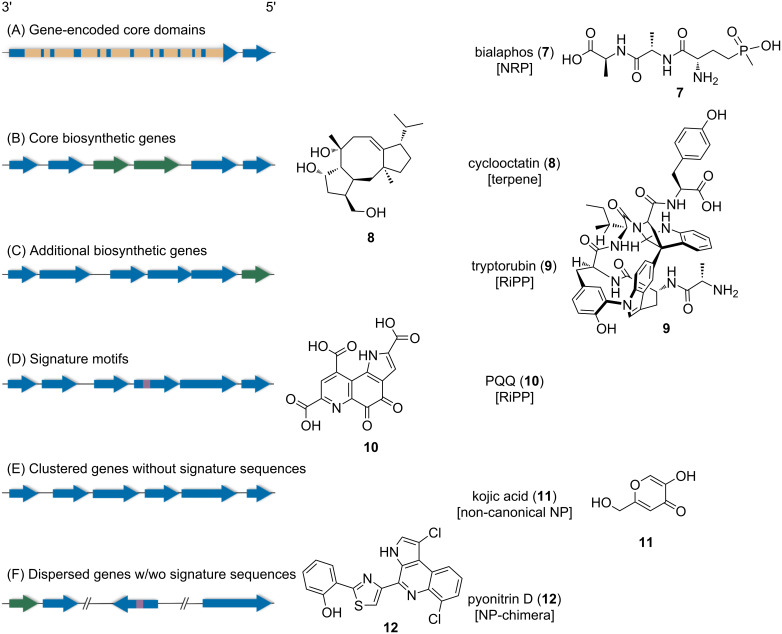
Universal (A, B, F) NP class and NP family-specific (C, D, F) signature sequences in NP BGCs and selected examples of each scenario. Genome mining tools utilize signature sequences to identify NP BGCs derived from (A) gene-encoded conserved core domains of assembly line-like pathways (e.g., modular NRPSs/PKSs), (B) distinct core enzymes (e.g., terpene or type II PKS biosynthesis), (C) tailoring enzymes (e.g., characterized families of RiPP biosynthetic pathways), (D) as well as signature motifs (e.g., RiPP biosynthetic pathways that utilize tailoring enzymes containing RREs). (E) BGCs without signature sequences (e.g., NRPS-independent alkaloid biosynthesis) or (F) genomically dispersed (i.e., not clustered) genes (here also referred to as biosynthetic gene sets) are difficult to identify. The enzymes encoded in core biosynthetic genes are responsible for assembling the NP backbone; additional biosynthetic genes encode tailoring enzymes and other components of a pathway (transporters, regulators, immunity enzymes). Conserved domains are depicted in yellow, genes in green and motifs in violet.

Assembly line-like pathways are characterized by mega enzymes, which can be subdivided into modules. Each module is responsible for the incorporation (and/or processing) of one building block into the nascent product. A “textbook” extension module minimally harbors three core domains, responsible for the activation and loading, tethering, and condensation of building blocks and intermediates. The biosynthesis is directional and starts at the N-terminal module with the activation and loading of the first building block onto the assembly line (Figure 2A) [21]. The specificity of the activating domain determines the type of building block incorporated. The growing intermediate stays permanently bound to the assembly line until the final product is released at the C-terminal module. However, modules can also be skipped, used for the modification of the nascent NP rather than its chain extension, or utilized iteratively [22–23]. In textbook assembly line-like pathways, the architecture of the mega enzyme complex correlates with the product structure, a principle that is referred to as the colinearity rule [24]. Examples of these assembly line-like pathways are canonical type I *cis*-acyltransferase polyketide synthases (PKSs) and type A non-ribosomal peptide synthetases (NRPSs) (Figure 2A) [25–26]. The substrate specificity of the specificity conferring domains in each module can be predicted from the sequences of adenylation (A) (for NRPS [26]), acyltransferase (AT) (for *cis*-AT PKS [15]), or ketosynthase (KS) domains (in *trans*-acyltransferase PKS systems [19,27]). Moreover, in the large majority of cases, the gene order within a BGC reflects the order of the corresponding enzymes during the biosynthesis of the associated NP [19]. *trans*-AT PKSs are much more complex than *cis*-AT PKS systems as they harbor non-elongating modules, cryptic domains and seemingly superfluous domains. Moreover, they frequently employ a number of *trans*-acting modifying enzymes, are characterized by modules that are split between proteins and they often harbor non-canonical module architectures and cryptic domains [19,22]. As a result, the colinearity rule cannot be applied to predict *trans*-AT PKS-derived polyketide core structures [19]. Instead, it has been observed that the amino acid sequences of the ketosynthase domains in *trans*-AT PKSs correlate with their substrate specificity [27]. This correlation can be used for the prediction of *trans*-AT PKS-derived polyketide core structures and is referred to as the correlation rule [19]. All commonly occurring domains in assembly line-like NP biosynthetic pathways as well as their non-modular homologs (e.g., type II and III PKSs) show a high degree of sequence homology. For that reason, their sequence can be used by genome mining tools as universal signature sequences to identify the genes encoding the respective domains and the remaining genes of the BGC (Figure 3A and B (e.g., bialaphos (**7**) [11])) [28].

In contrast, discrete multi-enzymatic assemblies utilize distinct, monofunctional enzymes. Examples are terpene (e.g., cyclooctatin (**8**) [29]), ribosomally synthesized and post-translationally modified peptide (RiPP), or NRPS-independent alkaloid pathways. In the case of terpene biosynthesis, terpene cyclases generate the oftentimes multicyclic, hydrocarbon scaffold via a carbocation-mediated cascade reaction [30]. Terpene cyclases are obligatory components of canonical terpene pathways and are used to identify terpene BGCs (Figure 3B) [30–31]. RiPPs, on the other hand, lack genes that are conserved across all 40 plus RiPP families [32]. However, each RiPP BGC family features genes encoding characteristic tailoring enzymes, or precursor peptides, that show a high degree of sequence conservation within the family. These conserved genes can be utilized for the targeted, family-specific identification of RiPP BGCs (Figure 3C (e.g., tryptorubin (**9**) [33])) [21]. In addition, multiple RiPP tailoring enzymes harbor a precursor peptide-binding domain, the so-called RiPP recognition element (RRE) (Figure 3D (e.g., pyrroloquinoline quinone (PQQ, **10**) [34])). RRE-derived signature motifs (i.e., short sequences that are conserved across different types of enzymes and that have a specific function) are used to identify RiPP BGCs beyond family borders as they are present in the BGCs of approximately 50% of all RiPP families [35]. BGCs without conserved signature sequences are almost impossible to identify using current bioinformatic approaches (Figure 3E (e.g., kojic acid (**11**) [36])). Therefore, the prediction of these BGCs is mainly based on the co-localization of adjacent genes encoding tailoring or additional core enzymes.

The current BGC prediction approach has its limitations, as genes involved in the biosynthesis of a NP might be dispersed (i.e., not clustered) throughout the genome and hence cannot be recognized by genome mining algorithms due to the missing proximity of the biosynthetic gene sets (BGSs) (Figure 3F (e.g., pyonitrin (**12**) [37])). NPs whose biosynthesis significantly deviates from the well-established biosynthetic principles (e.g., through the lack of signature sequences) (Figure 3E) [38] are frequently overlooked by state-of-the-art genome mining pipelines. Most genome mining algorithms rely on the identification of signature sequences (Figure 3A–D). As a result, BGCs of the most commonly studied NP classes (e.g., PKS and NRPS BGCs) can be identified with high confidence based on the sequence homology of the commonly occurring biosynthetic domains. Since chemical novelty in assembly line-like pathways is typically obtained through novel arrangements of a limited set of module architectures, a limited diversity of sequential module arrangements, and varying substrate specificities, the probability of identifying truly novel biosynthetic principles and biochemical transformations in these systems is restricted when using hard-coded biosynthetic principles that are based on the detection of the frequently encountered biosynthetic domains [21]. As a result, a lot of effort is currently being put into the development of complementing workflows to chart the “biosynthetic dark matter” (i.e., overlooked biosynthetic pathways) that we currently cannot access bioinformatically [39]. State-of-the-art genome mining tools are ideally suited for the detection of assembly line-like pathways. The focus on these pathways led to a strong bias in training sets: In the MIBiG database of characterized BGCs nearly 80% of all deposited NP BGCs are PKS, NRPS, or terpene BGCs (April 2022) [20]. As the largest database of characterized BGCs, MIBiG is frequently used as a training data set for the development of genome mining algorithms. The imbalanced representation of NP BGCs in the database, however, might introduce a bias when it comes to the training of novel algorithms. Another obstacle to overcome is the efficient mining of the vast quantity of genomic data generated via next-generation sequencing, as a lot of genome mining algorithms are not capable of handling big data [40].

### Genome mining principles and tools

Many genome mining tools are based on gene homology and rely on alignments of annotated open reading frames (ORFs). Yet, their purpose, functions, and additional features such as comparative analyses of BGCs, dereplication concepts, or NP structure prediction differ significantly. In addition, some tools implement alternative BGC identification methods like phylogenetic analyses or ML approaches. In many cases, these ML approaches are based on well-established strategies adopted from other disciplines (e.g., natural language processing or comparative genomics) that were adapted by the NP community [41].

In the following section, we will look at representative genome mining tools and discuss their underlying BGC detection principles, along with advantages and limitations of the BGC identification process.

### Genome mining algorithms based on hard-coded biosynthetic principles

**Figure 4:**
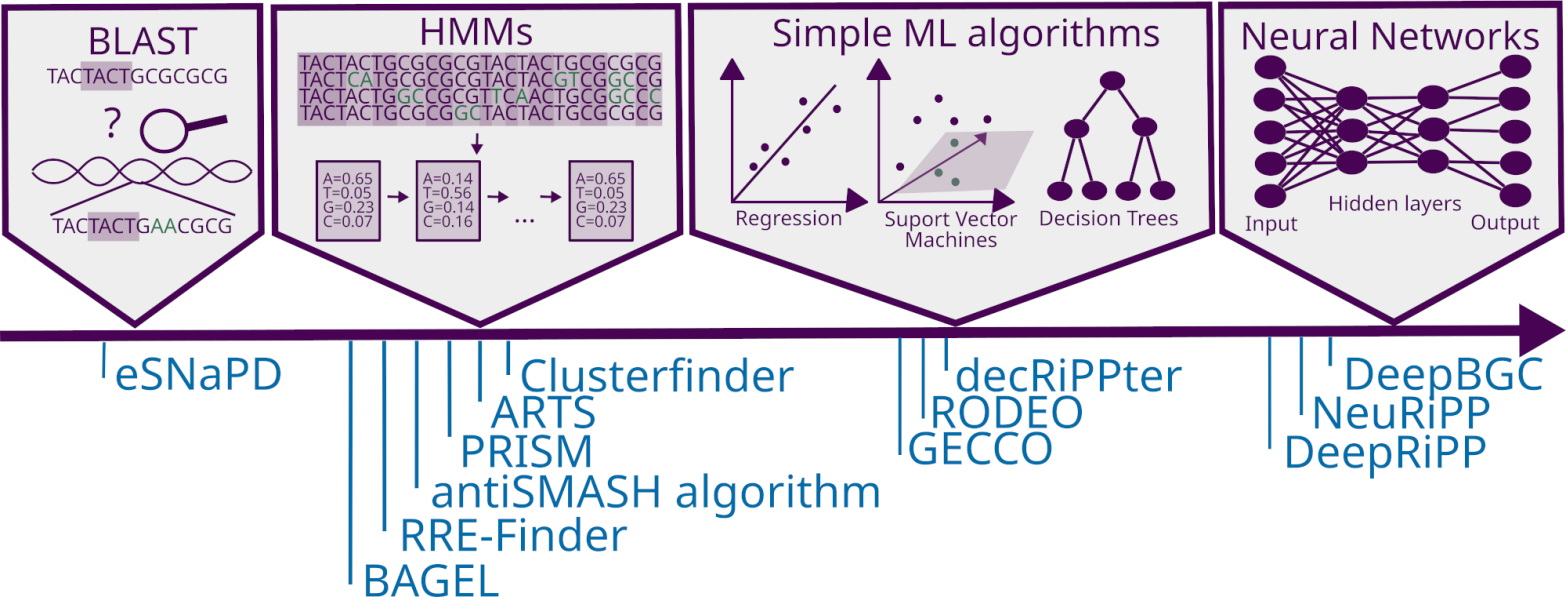
Concepts of algorithms in order of complexity and examples of genome mining tools that employ the respective concept.

An early approach to identify NP BGCs in (meta-)genomic data sets were sequence alignments with known genes and domains using algorithms like BLAST (Figure 4) [42]. BLAST detects similar sequences to a given query sequence [42]. The first version of the tool BAGEL utilized BLAST analysis, among others, to identify putative BGCs of bacteriocins (= antimicrobial peptides and proteins) [43–46]. The advantage of such reference alignment methods that are based on sequence homology is their high confidence. The performance of these tools can be rapidly improved via the addition of new reference databases, which was contributing to their success at the beginning of the genome mining era. However, using BLAST-based approaches, the identification of real structural or biosynthetic novelty remains relatively sparse, as the BLAST algorithm is most suitable to detect close homologs of the query sequence. Up to this day, tools like BAGEL are predestined for the rapid and computationally cost-effective characterization of genomic data [43].

Hidden Markov Models (HMMs) are statistical models that are used by the NP community as a more flexible approach to identify BGCs (Figure 4). These models consist of a sequence of “states” (e.g., the occurrences of specific amino acids or nucleotides at a certain position of a protein or DNA sequence, respectively) with pre-determined transition probabilities from one state to the next (e.g., the transition probability in a sequence between one base at a given position to another base at the next position). A sequence of probabilities is calculated from given sequence alignments, for instance, of members of a given gene or protein family. By adding up all possibilities, the likelihood of the complete sequence being a member of the gene family can be calculated [47]. Derivatives of HMMs, so-called profile Hidden Markov Models (pHMMs), are additionally taking gaps and incomplete sequences into consideration. In addition to whole genes or proteins, sequences of conserved key domains of assembly line-like pathways like PKSs (e.g., acyl-carrier-proteins, AT or KS domains) [25] or NRPSs (e.g., peptidyl-carrier-proteins, A domains, condensation (C) domains) [26] are utilized for the generation of pHMMs. The resulting pHMMs recognize signature sequences of such conserved domains in genomic query sequences. pHMMs cannot only be employed to detect and annotate BGCs but also to predict substrate specificities that are essential for NP structure predictions [19,39,48]. After the identification of the core biosynthetic genes, co-localized genes are analyzed and the locus and borders of the BGC are predicted via hard-coded rules based on textbook biosynthetic knowledge, e.g., the minimum amount of domains in a typical NRPS. Due to their seemingly universal biosynthetic principles and modular composition, canonical PKS and NRPS BGCs are predestined for the high confidence detection of their encoded biosynthetic core domains using pHMMs. Structural novelty in these systems that predominantly comprise the same set of conserved domains arises from the novel arrangement of the limited set of different module architectures (e.g., around a dozen in *cis*-AT PKSs vs >150 in *trans*-AT PKSs [19]) along with varying substrate selectivities of specificity-conferring domains (e.g., A domains in NRPSs, AT domains in *cis*-AT PKSs, and KS domains in *trans*-AT PKSs). Moreover, since these assembly line-like pathways follow the same biosynthetic principle, they often form hybrids with other biosynthetic assembly line-like pathways [21].

Prominent examples of the usage of pHMMs are the original algorithm of the antibiotics & Secondary Metabolite Analysis Shell (antiSMASH) [17,29,49–52] as well as PRediction Informatics for Secondary Metabolomes (PRISM) [18,53–55]. In addition to PKSs and NRPSs, both tools identify a high number of NP classes and families using pHMMs (antiSMASH 6: 876 pHMMs, PRISM 4: 1772 pHMMS). Apart from BGC detection by pHMMs, several stand-alone tools have been implemented into antiSMASH to improve BGC identification, annotation, and substrate predictions (Table 1) (described in detail below). Therefore, we distinguish the original antiSMASH algorithm from the antiSMASH platform (Table 1). Although the BGC identification approach of antiSMASH and PRISM is quite similar, both tools differ in the downstream processing of the identified BGCs. While antiSMASH focuses on functional and comparative analyses of the biosynthetic genes and BGCs, the focus of PRISM lies on a comprehensive chemical structure prediction of the associated NP [56–58]. In silico dereplication to eliminate BGCs associated with known NPs is one of the major functions of genome mining to avoid the time-consuming and costly re-isolation of known NPs. For instance, the antiSMASH platform compares putative BGCs with reference databases to detect BGCs that are similar to previously characterized BGCs [15,20,58]. However, as many NPs were isolated during the pre-genomic era, they have not been linked to their corresponding BGC. As a result, BGC databases are incomplete which is a drawback when it comes to the dereplication on a gene level. PRISM aims at overcoming this obstacle via retro-biosynthetic building block predictions of known NPs from multiple databases in combination with several BGC-derived NP structure suggestions [58].

**Table 1:** Purpose, principles, advantages, and disadvantages of selected genome mining tools. The upper part of the table contains hard-coded tools and the lower part ML-based tools. Novelty refers to the ability of a genome mining tool to chart non-canonical BGCs. Confidence refers to the ability of a genome mining tool to correctly identify a NP BGC.

Tool [first/latest version]	Purpose	BGC identification principles	(Dis-)advantages	Novelty	Confidence

antiSMASH algorithm [17] 2011/2021	identification of a broad range of NP BGC classes and families	pHMMs, hard-coded rules	comprehensive NP class detection	low	high
					
antiSMASH platform [17] 2011/2021 https://antismash.secondarymetabolites.org	identification of a broad range of NP BGC classes and families, functional and comparative analyses, structure prediction	ClusterFinder^a^: pHMM RODEO: BLAST, pHMM, SVMs RRE-Finder: pHMMs/HHpred database	comprehensive analysis covering many NP classes, dereplication via comparative analysis, usage of NP BGC databases	medium	high
					
ARTS [59] 2017/2020 http://arts.ziemertlab.com/index	target directed genome mining for antibiotics in bacteria via resistance genes	pHMMs for BGC prediction (antiSMASH), TIGRFAM for detection of housekeeping genes, phylogenetic analysis for identification of horizontal gene transfer	targeted approach for bioactivity	low	high
					
BAGEL [49] 2006/2018 http://bagel4.molgenrug.nl/	identification of bacterial bacteriocins and RiPPs in (meta-) genomic sequences	BLAST analysis, HMMs, hard-coded rules	restricted to RiPP and bacteriocin BGCs	low	high
					
CASSIS and SMIPS [60] 2016 https://sbi.hki-jena.de/cassis/	BGC detection in fungi	CASSIS: Density of transcription factor binding sites, SMIPS: Signature sequences	precise cluster borders	low	high
					
ClusterFinder [61] 2014 Implemented in antiSMASH	BGC detection without functional assignment of NP class	HMM for whole cluster	comprehensive NP class detection	high	low
					
eSNaPD [62] 2014 http://esnapd2.rockefeller.edu/	BGC detection in non-assembled bacterial metagenomic sequences	BLAST analysis against BGC database	comprehensive NP class detection of smaller BGCs that are similar to known BGCs	low	high
					
EvoMining [63] 2016/2019 https://github.com/nselem/evomining	identification of BGCs integrating evolutionary principles	phylogenomic analysis in combination with antiSMASH analysis	independent of commonly used signature sequences	medium	medium
					
PRISM [55] 2015/2020 https://prism.adapsyn.com/	identification of a broad range of NP BGCs, structure prediction	HMMs for BGC detection, BLAST analysis, protein motifs and HMMs for domain specificity prediction, support vector machines for activity prediction	comprehensive analysis covering many NP classes, several structure suggestions, dereplication via structural comparisons	low	high
					
SMURF [64] 2010 http://smurf.jcvi.org/run_smurf.php	identification of fungal BGCs	HMMs, hard-coded rules	comprehensive NP class detection	low	high

transATor [19] 2019	annotation of *trans*-AT PKSs and accurate structure predictions of *trans*-AT PKS-derived polyketides	pHMMs, hard-coded rules	restricted to *trans*-AT PKSs	low	high

decRiPPter [65] 2020 https://github.com/Alexamk/decRiPPter	identification of RiPP BGCs	SVMs, pan-genomic analyses	restricted to RiPP BGCs	medium	medium
					
DeepBGC [41] 2019 https://github.com/Merck/deepbgc	identification of bacterial and fungal BGCs	neural network with vector- represented Pfam domains (ML)	comprehensive NP class detection	high	medium
					
DeepRiPP [66] 2019 http://deepripp.magarveylab.ca	identification of RiPP BGCs, structure prediction	natural language processing (deep learning)	restricted to RiPP BGCs	medium	medium
					
GECCO [67] 2021 https://github.com/zellerlab/GECCO	identification of bacterial and fungal BGCs	conditional random fields	comprehensive NP class detection	high	medium
					
NeuRiPP [68] 2019 https://github.com/emzodls/neuripp	identification of RiPP precursors	neural networks	restricted to RiPP precursors	medium	medium

RODEO [69] 2017 https://rodeo.scs.illinois.edu/	identification of RiPP BGCs	BLAST analysis of tailoring enzymes, pHMMs, SVMs for precursor detection	restricted to RiPP BGCs	low	medium

^a^ClusterFinder is not available on the antiSMASH web server any longer but is incorporated into the standalone antiSMASH command line tool.

To identify RiPP BGCs, the antiSMASH algorithm and PRISM utilize pHMMs based on RiPP-family-specific signature sequences derived from tailoring enzymes or precursor peptides (Figure 3C and D). These family-specific pHMMs are likewise used in tools like BAGEL or RODEO and enable the identification of novel members of known RiPP families [46,69]. RRE-Finder, which is integrated into the antiSMASH platform and RODEO, utilizes the presence of RREs, predicted via pHMMs, to detect RiPP BGCs (Figure 3D). Since the RRE motif is only present in approximately 50% of all RiPP families, it restricts the predictable biosynthetic space. Yet, RRE-Finder is one of the few RiPP genome mining tools which is capable of identifying RiPP BGCs in a family-independent manner [70].

Since the potential of identifying truly novel BGCs via signature sequences is limited, the tool ClusterFinder was developed and implemented into the command line version of antiSMASH [61]. ClusterFinder annotates BGCs via pHMMs from a string of contiguous Pfam domains (protein domains annotated in the protein family database) instead of individual genes. pHMMs are calculated using training sets of known BGCs and non-BGC sequences. Here, two states “BGC” and “non-BGC” are distinguished depending on the Pfam domain frequency in the training data set and the identities of adjacent domains. Consequently, the ClusterFinder algorithm is designed to detect BGCs that are overlooked by other biosynthetic pipelines. As in many other algorithms for the detection of true biosynthetic novelty, high false positive rates have to be taken into consideration, which makes the output of low-confidence/high novelty algorithms more difficult to interpret [61].

An alternative to the above mentioned classical genome mining approaches is the utilization of evolutionary information for the detection of NP BGCs. The EvoMining concept is based on the assumption that secondary metabolite biosynthetic enzymes are distant paralogs of enzymes involved in primary metabolism [63,71]. These NP biosynthetic enzymes are hypothesized to have undergone significant sequence and selectivity changes while still operating based on the same reaction mechanism (e.g., fatty acid biosynthesis → polyketide biosynthesis). As such, NP biosynthetic pathways utilize members of existing enzyme families that have evolved to perform new metabolic functions. Consequently, NP BGCs “borrow” genes encoding paralogs of enzymes that have their origin in primary metabolism and that have diverged into catalyzing alternative metabolic functions. That way, the EvoMining approach identifies members of biosynthetic enzyme families that have likely been repurposed and thus, their corresponding genes are prime targets for a closer inspection of the genomic context to identify new types of BGCs. Although EvoMining is a signature sequence independent concept and instead uses phylogenetic analysis of primary metabolite biosynthetic enzymes, it remains a “hard-coded” sequence similarity-based approach that uses phylogenetic analysis instead of pHMMs for BGC detection [63,71].

### Machine learning-based genome mining tools

Some NP BGCs contain solely family-specific features, and lack universal class-specific signature sequences. In these cases, only members of the same subfamily can be identified via pHMMs. An example of the latter are RiPPs that are the most rapidly expanding NP subclass. Eighteen new RiPP families have been characterized over the span of just 8 years, suggesting that many more RiPP families have yet to be discovered [32]. To exploit these currently overlooked biosynthetic treasures, multiple recently developed genome mining tools make use of ML algorithms that have been adapted from other research fields like image recognition [65–68]. Most ML-based tools utilize “supervised learning,” a strategy that employs a dataset with known classifications to train the algorithm [72]. Traditional ML algorithms include regression, decision tree-based classifiers, and support vector machines (SVM), which construct a hyperplane that splits the *n*-dimensional data-space (i.e., different features/categories serve as dimensions of this space) into different areas that correspond to the different classes (Figure 4) [72]. These algorithms usually lead to robust and interpretable predictions but are limited when it comes to solving complex problems [72].

An example of an advanced combination of different approaches and methods for the identification of RiPPs is the Data-driven Exploratory Class-independent RiPP TrackER (decRiPPter) [65]. decRiPPter uses a support vector machine algorithm trained on a set of known precursor genes to detect RiPP precursor genes semi-independently of their subclass. Subsequently, a pan-genome analysis is performed to identify the corresponding BGCs with the putative RiPP precursor genes as seeds. Putative NP BGCs are identified that are organized in operon-like structures and prioritized based on the taxonomic distribution of the cluster. decRiPPter was successfully used for the identification of a new lanthipeptide subfamily, providing experimental validation of the algorithm [65].

A more advanced form of supervised learning is deep learning (Figure 4). An example of a deep learning architecture is the artificial neural network inspired by the human brain architecture. It consists of artificial neurons processing information organized in different layers and connected by synapses [73]. These advanced algorithms often provide higher accuracy in their prediction but are no longer interpretable as a result of their high level of abstraction [73]. NeuRiPP, for instance, utilizes a parallel convolutional neural network to predict novel RiPP precursor genes independent of their RiPP family. The neural network is trained on a RiPP precursor training set that is based on experimentally verified precursors and precursors predicted by other tools [68]. Both RiPP-specific tools, NeuRiPP and decRiPPter, allow a more flexible BGC identification than hard-coded algorithms but are biased in that the precursor identification depends on training sets consisting of precursors from known RiPP families.

In contrast to NeuRiPP and decRiPPter, DeepBGC is not restricted to a single NP class. Comparable to some hard-coded algorithms, DeepBGC is based on HMM-generated Pfam annotations. However, instead of utilizing Pfam-domains as features, it converts the arrays of Pfam annotations into numeric vectors using a shallow two-layer neural network, an approach adopted from natural language processing [41,72]. These high-dimensional vectors are then used as input for a second two-layer neural network trained on a set of BGC and non-BGC sequences to predict NP BGCs. In the last step, the NP class is predicted using a random forest classifier (Figure 4) [41,74]. DeepBGC outperforms ClusterFinder in its accuracy and false-positive rates due to its ML approach. Like with many other tools, a major disadvantage of DeepBGC is that BGCs lacking canonical biosynthetic domains and small BGCs are filtered out in a pruning stage. Consequently, small BGCs (e.g., biarylitides **15** [75] and tryptorubins **9** [33]) or those that feature solely atypical biosynthetic genes are not recognized, which reduces the likelihood of identifying true biosynthetic novelty [41].

A similar approach is utilized by GECCO, that uses conditional random fields on arrays of Pfam annotations [67]. Conditional random fields belong to the statistical methods and can be classified between HMMs and simpler machine learning algorithms. An advantage of conditional random fields is their interpretability [67]. GECCO outperforms rule-based models in terms of novelty and DeepBGC in terms of accuracy while being less computationally expensive than both [67]. Like DeepBGC, GECCO currently lacks functional proof for the identification of a novel natural product guided by the tool [67].

### Challenges and potential solutions to identify currently overlooked BGCs

**Figure 5:**
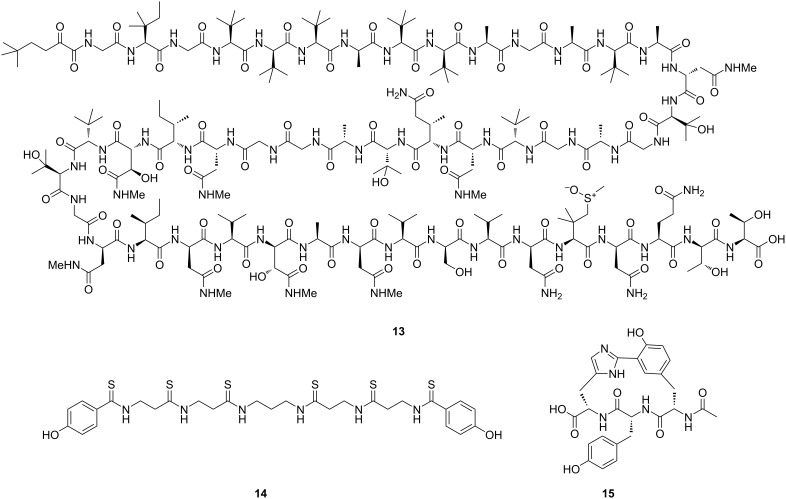
Examples of peptide NPs, the corresponding BGCs of which were determined through retrobiosynthetic analysis and then experimentally verified: polytheonamide A (**13**) [76], closthioamide (**14**) [77], and biarylitide YYH (**15**) [33].

Genome mining was pivotal for the expansion of NP chemical space in the past two decades. Despite the development of more and more sophisticated genome mining platforms, in many cases where truly novel NP scaffolds were described, the NP was isolated first and only then linked to its corresponding BGC [38]. Notable examples include the (thio-)peptides polytheonamide A (**13**) [76], closthioamide (**15**) [77] (Figure 5), and tryptorubin A (**9**) [33]. It was not until the structure of each of these peptides was determined, that manual retrobiosynthetic analysis resulted in the proposal of biosynthetic models that were subsequently experimentally verified. Once the biosynthesis of a NP is determined using this approach, the NP family can be expanded by developing genome mining algorithms to identify BGCs that follow similar biosynthetic principles [56].

One crucial challenge in the development of novel genome mining tools is balancing novelty and confidence, as one tends to fall short as the other is optimized [39]. On the one hand, genome mining tools that are focused on detecting non-canonical BGCs (high-novelty) are usually characterized by the identification of many putative BGCs that might not be involved in NP biosynthesis (high false-positive rate). These false-positive BGCs are automatically pruned and the resulting putative BGCs need to undergo a second round of manual verification and prioritization prior to functional characterization [39]. On the other hand, hard-coded algorithms detect BGC with high confidence but are restricted when it comes to the identification of BGCs that deviate significantly from what the algorithm's pHMMs have been trained to identify (true biosynthetic novelty) [39]. As most algorithms are at least to some extent signature sequence or sequence homology based, they heavily rely on the sequence space of known BGCs. The bias of hard-coded algorithms is embedded in the biosynthetic rules used for BGC detection and the dataset used to create pHMMs. The bias of ML-based algorithms results from their training sets that usually consist of characterized, canonical BGCs that are then used for the targeted identification of non-canonical BGCs [39]. Utilizing fewer gene family-based features, like the occurrence of Pfam domains or the sequence itself, for predictions can help to avoid overfitting, i.e., the problem of getting the algorithm to perform very well on the training data but underperform on unseen data [39].

Most genome mining algorithms rely on functionally annotated ORFs for the prediction of BGCs. State-of-the-art genome annotation algorithms are not yet able to recognize all ORFs correctly, especially very short ORFs like RiPP precursor genes [78]. Combined with many false ORF annotations, missing annotations impair BGC predictions downstream of the annotation process. Moreover, the BGCs of certain NP families are inherently easier to identify than others. For example, domains of canonical NRPSs and PKSs can be identified by signature sequence-based pHMMs with high confidence (Figure 3). Furthermore, pHMMs of conserved domains can be subdivided into dozens of individual pHMMs used to determine the substrate specificity of a conserved domain [17,19]. However, BGCs lacking known signature sequences are inherently more difficult to identify. In addition, the size of the BGC of interest impacts the predictive power of the algorithms: Extremely small BGCs, harboring only a few genes, are frequently overlooked as they usually do not pass hard-coded thresholds. For instance, the 1.2 kb gene cluster linked to tryptorubin (**9**) biosynthesis only encodes a 26 amino acid precursor peptide and a single cytochrome P450 monooxygenase [33,79], and hence it was overlooked by genome mining algorithms. On the other hand, large PKS or NRPS BGCs can be split across multiple contigs. This mosaic-like distribution of a single BGC makes the identification of the entire BGC a challenging endeavor especially if multiple assembly line-like BGCs are present in a genome. Moreover, the quality of assembled genomes obtained from short reads decreases with highly repetitive sequences present in many large PKS or NRPS genes [39].

Although the traditional hard-coded rule- and ML-based approaches differ fundamentally when it comes to the implementation of the respective NP BGC identification, they are both based on the same principle: The direct identification of NP BGCs. Both approaches heavily rely on training sets to generate pHMMs or to train the respective ML algorithm. As a consequence, they are both hypothesis-driven approaches resulting in an inherent bias “to identify what the algorithm was trained to identify” rather than to chart the entire biosynthetic space. This bias is largely based on the fact that both approaches use the characterized NP biosynthetic space as a training set for its expansion. Even though there might be no truly unbiased approach towards the expansion of NP biosynthetic space, indirect NP BGC detection methods might be capable of complementing the current strategies. These indirect approaches are exclusively based on the assumption that NP biosynthetic genes are clustered in microbial genomes (even though this might not be true for all NP biosynthetic pathways) and do not require prior knowledge about characterized biosynthetic pathways as training data sets. Below, we are showcasing two putative solutions to complement existing approaches to expand NP biosynthetic space and to chart biosynthetic dark matter.

### Genome-wide characterization of all clustered genes as an approach to identify non-canonical pathways

**Figure 6:**
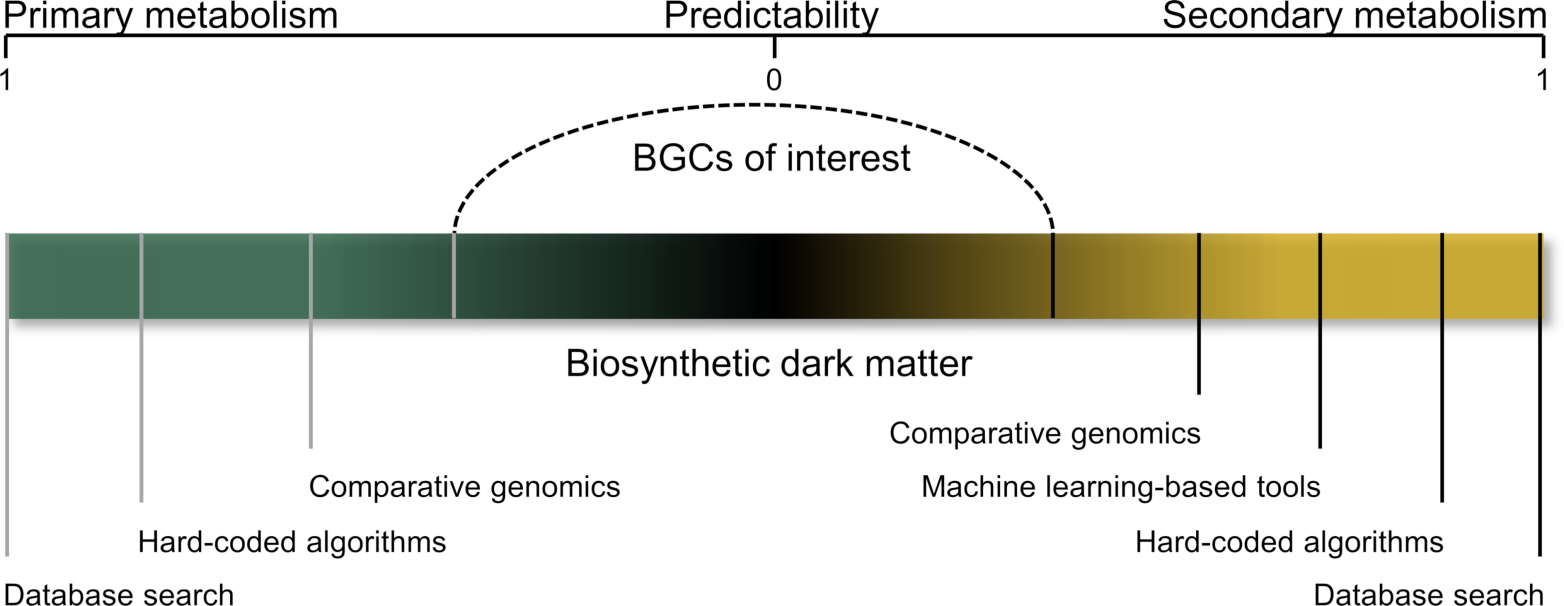
gcBGC prioritization process. The bar represents all identified gene clusters in one organism and is subdivided into primary (green) on the left and secondary metabolism (yellow) on the right. Gene clusters associated with primary or secondary metabolism (both ends of the spectrum) are identified using the strategies listed below the bar and subsequently excluded from further analysis. gcBGC aims at identifying as-of-yet overlooked BGCs that cannot be detected by state-of-the-art bioinformatic platforms, here referred to as “biosynthetic dark matter.”

One concept that is based on the above outlined indirect approach is the genome-wide characterization of all clustered genes (gcBGC). In comparison to state-of-the-art genome mining tools, gcBGC inverts the current BGC identification process. Instead of identifying NP BGCs, all clustered genes involved in primary and secondary metabolite biosynthesis are identified. To specifically target non-canonical BGCs, BGCs that can be unambiguously assigned to primary metabolism and those BGCs that are detected by state-of-the-art genome mining pipelines are filtered out. Based on the initial hypothesis underlying the gcBGC approach, the remaining BGCs are likely involved in non-canonical NP biosynthesis (“biosynthetic dark matter” in Figure 6).

The gcBGC concept is based on the assumption that secondary metabolite BGCs evolve from primary metabolite biosynthetic pathways, and that the transition between both is fluid [71]. First, gcBGC identifies all clustered genes in a signature sequence-independent manner via analysis of operon-like structures (e.g., promoters or transcription start sites) as shown in fungi by the tool CASSIS/SMIPS [60]. This concept contrasts the commonly used principles that rely on the direct detection of genes via (p)HMMs- or ML-based approaches, both of which typically require a training data set.

As this approach leads to the identification of a large number of primary and secondary metabolite BGCs that are likewise detected by state-of-the-art genome mining pipelines, a filtering step is required to prioritize the putative non-canonical BGCs that are currently overlooked by existing genome mining tools [17,41,55,80] (Figure 6). Moreover, additional information on taxonomic relationships, pan-genome analyses, or whole-genome comparisons of all members of the pan-genome can be used for further prioritization (Figure 6) [81]. gcBGC is restricted to well-studied organisms where primary metabolite gene cassettes can be confidentially identified. However, the inverted BGC identification concept combined with the focus on as-of-yet unidentified BGCs suggests gcBGC-like approaches to be promising alternatives for the detection of non-canonical pathways.

### A comparative genomics approach to identify non-canonical BGCs

**Figure 7:**
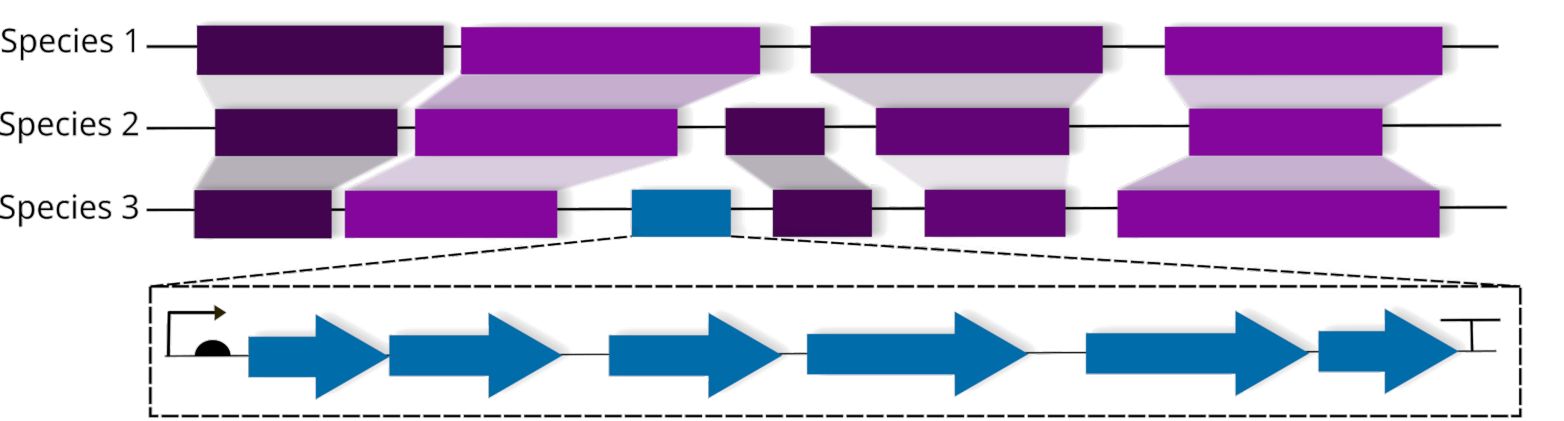
Genome alignments of related organisms revealing the presence of syntenic (purple) as well as non-syntenic blocks (blue). The order of syntenic blocks can be disrupted by putative HGT events, leading to the integration of non-syntenic blocks that are subsequently screened for operon-like structures containing multiple continuous gene arrangements (blue) without large gaps.

Another concept for the expansion of NP biosynthetic space is based on a Comparative Genomics Approach (CGA). This approach relies on the fact that many BGCs are introduced into microbial genomes via horizontal gene transfer (HGT). A genome can be subdivided into groups of genes called syntenic blocks [82]. Among related strains, the order of these syntenic blocks, as well as their gene composition, is highly similar (Figure 7). Evolutionary young HGT events in single/few strains can disrupt this order, leading to the insertion of non-syntenic blocks (Figure 7) [82]. These insertions are detectable by comparing multiple closely related strains utilizing whole genome alignments, a technique adopted from the field of comparative genomics [83]. In a recent study, 10 *Aspergillus* genomes were compared to identify BGCs in non-syntenic blocks, leading to the confirmation of all previously known BGCs using the CGA concept [84]. As a proof of concept, the previously characterized kojic acid (**11**) BGC, which escaped detection by state-of-the-art genome mining algorithms, was identified [84]. The kojic acid (**11**) BGC lacks the classical biosynthetic signature sequences typically used for BGC identification, thus showing the potential of the approach (Figure 3) [84].

CGA aims at scaling this approach and comparing all sequenced strains of one genus (e.g., *Streptomyces*) to find non-syntenic blocks that might code for NP BGCs. Comparable to the genome-wide characterization of all clustered genes concept, CGA focuses on BGC detection independently of signature sequences and known NP families to expand the known NP chemical space via the identification of non-canonical pathways.

The first step of CGA consists of the homogenous functional annotation of all genes of the selected genomes to reduce false positive rates of non-syntenic blocks due to different annotations of genes using different annotation algorithms. Subsequently, all annotated genes are clustered based on sequence similarity to improve functional annotations [85]. The obtained sequential arrangements of gene annotations representing the different genomes are aligned to compare the genomes not on a sequence level, but instead on the gene-function level [86].

Whole genome alignments are performed to detect single diverging gene loci that are subsequently expanded by their genomic neighborhood to detect genomic islands. Therefore, the genomic neighborhoods of the identified genes are analyzed for differences in their synteny to detect HGT regions composed of multiple genes. In addition, these regions are analyzed for genetic characteristics like promoters or transposase genes to identify operon-like structures. Single gene duplication events are filtered out and all known BGCs are excluded in a similar fashion as in the gcBGC approach. The presence of prototypical tailoring enzymes ubiquitously distributed in secondary metabolism might serve as an additional line of evidence for a functional NP BGC. This approach is computationally expensive yet feasible with the availability of high-performance computer clusters but requires excellent quality of the analyzed genomes. This method has the advantage over simpler approaches to detect HGT events, like for example comparing GC contents of different regions, that it can be used to detect HGT events from closely related strains.

## Conclusion

The development of next-generation sequencing technologies [4] and the resulting availability of a seemingly exponentially increasing number of genome sequences enabled or revolutionized several biological fields including comparative genomics [81], functional genomics [87], and NP-genome mining [88]. From simple BLAST analyses through pHMM-based algorithms to ML-based approaches, genome mining is a continuously evolving field that has benefited from other disciplines, such as mathematics, image processing, or linguistics. State-of-the-art sequence homology- and ML-based genome mining tools identify BGCs that share even low levels of similarity with known BGCs with high confidence. Traditional pHMMs-based approaches are ideally suited to chart the biosynthetic space of assembly line-like pathways that are typically composed of novel arrangements of recurring module architectures with varying specifications of the substrate specificity-conferring domains. ML-based approaches on the other hand are more frequently employed to target non-homogeneous NP classes such as RiPPs whose BGCs do not share sequence homologies across all 40 plus RiPP-families and to identify NP BGCs that are currently overlooked by state-of-the-art sequence homology-based tools. Even though the scope and implementation of both approaches differs significantly, the underlying concept is the same: The direct, hypothesis-driven identification of clustered NP biosynthetic genes based on a training data set that requires a database of characterized BGCs. This training data set might comprise individual domains from characterized pathways to generate pHMMs to complex features that are extracted from characterized BGCs. The bias introduced through the dependence on these reference datasets is likely to result in an inherent limitation when it comes to the identification of truly non-canonical pathways that share low to no similarity to characterized pathways. To address these limitations, indirect approaches that do not rely on training data sets of characterized BGCs might be capable of complementing the current suite of highly sophisticated genome mining tools as they might be ideally suited to identify non-canonical pathways that are overlooked by direct identification approaches. We showcased two such hypothetical indirect approaches that we named “genome-wide characterization of all clustered genes” and “comparative genomics-based identification of non-canonical BGCs”. These indirect BGC detection concepts are solely based on the assumption that biosynthetic genes are clustered in bacterial genomes. Both approaches are based on the sequence similarity-independent identification of non-canonical BGCs via recognition of operon-like structures or usage of comparative genomics to detect horizontally transferred gene clusters. In a subsequent prioritization step, clustered genes that are involved in primary metabolite biosynthesis or that can be likewise detected by state-of-the-art genome mining pipelines can be excluded to target uncharted biosynthetic space also referred to as biosynthetic dark matter. These indirect concepts might serve as an inspiration for further innovative tools for the targeted discovery of hidden biosynthetic treasures.
